# Probiotics and Honey: Boosting Functional Properties in Dry Fermented Sausages

**DOI:** 10.3390/microorganisms13020349

**Published:** 2025-02-06

**Authors:** Tanja Žugić Petrović, Vladimir M. Tomović, Katarina G. Marković, Teresa Semedo-Lemsaddek, Mirjana Ž. Grujović

**Affiliations:** 1 Bio Food Viking, Karadjordjeva bb, 18230 Sokobanja, Serbia; tanja.zugicpetrovic@yahoo.com; 2 Faculty of Technology, University in Novi Sad, Cara Lazara 1, 21000 Novi Sad, Serbia; tomovic@uns.ac.rs; 3 Department of Science, Institute for Information Technologies, University of Kragujevac, Jovana Cvijica bb, 34000 Kragujevac, Serbia; katarinam@kg.ac.rs; 4 Centre for Interdisciplinary Research in Animal Health (CIISA), Faculty of Veterinary Medicine, University of Lisbon, Av. da Universidade Técnica, 1300-477 Lisbon, Portugal; 5 Associate Laboratory for Animal and Veterinary Sciences (AL4AnimalS), 1300-477 Lisbon, Portugal; 6 BioISI—Biosystems & Integrative Sciences Institute, Faculty of Sciences, University of Lisbon, 1749-016 Lisbon, Portugal

**Keywords:** dry-fermented sausages, Sokobanja sausage, starter cultures, lactic acid bacteria (LAB), sunflower honey, functional food

## Abstract

Dry-fermented sausages, particularly traditional varieties like Sokobanja sausage from Serbia, are highly valued for their unique sensory attributes. This study aimed to evaluate the effects of adding starter cultures (lactic acid bacteria, LAB, and coagulase-negative staphylococci, CNS) and organic sunflower honey (at concentrations of 0.2% and 0.4%) on the physicochemical, microbiological, and sensory properties of Sokobanja sausage. The primary objective was to enhance the sausage’s quality while accelerating the ripening process. The methodology involved enriching the sausage mixture with starter cultures and honey, followed by sensory evaluation, microbiological analyses, and physicochemical measurements over a 28-day ripening period. Results showed that the addition of starter cultures and 0.2% honey significantly improved texture parameters such as hardness, cohesiveness, and chewiness compared to the control. Consumer acceptance was also high for these sausages. Microbiological analysis revealed that honey supported the growth of LAB and CNS, which facilitated lactic acid production and resulted in a rapid decline in undesirable microorganisms, such as enterobacteria, yeasts, and molds, particularly after 7–14 days. This led to a reduction in pH and an accelerated ripening process, typically lasting 25–28 days. The findings suggest that incorporating starter cultures and sunflower honey enhances both the functional and sensory properties of Sokobanja sausage, offering a promising approach for improving quality and safety. Future research should explore the use of targeted delivery mechanisms for probiotic bacteria in the gastrointestinal tract and further investigate the potential health benefits of these sausages as functional foods.

## 1. Introduction

Meat fermentation has been used as a method of preserving meat products for centuries, particularly in sausage production [1]. Fermented sausage is made from minced meat mixed with spices, such as salt and sugar, and drying agents. The mixture undergoes fermentation and is then encased. Traditional sausages are increasingly valued by consumers for their unique sensory attributes. These sausages are often produced in local households through spontaneous fermentation using autochthonous bacterial cultures [2,3].

Serbia is renowned for its artisanal meat products, which have a long-standing tradition. Today, various regional variations of these products exist. Among the most popular traditionally fermented sausages in Serbia are “Pirot ironed” sausage, Petrovská klobasá, “Sremski kulen”, and “Sremska” sausage [4,5,6,7,8,9]. Petrovská klobasá, in particular, has been produced for generations using traditional techniques that exclude additives such as nitrates, nitrites, glucano-d-lactone, and starter cultures. It is characterized by its distinctively spicy taste, aromatic flavor, dark red color, and firm texture [4,5,7,10]. Due to its unique and recognizable quality, this sausage holds a Protected Designation of Origin (PDO) under Serbian legislation [4]. The quality of raw meat, processing conditions, and the microorganisms present in fermented sausages collectively determine the final product’s characteristics. Fermentation involves a complex microbial community of bacteria, yeast, and mold that plays a crucial role in transforming raw materials. This process enhances various physicochemical, sensory, and microbiological properties. While traditional methods rely on natural fermentation, commercial starter cultures are sometimes added to raw meat during production [2].

Starter cultures in meat products are defined as “viable microorganisms that multiply within meat products, enhancing preservation, ensuring hygienic safety, and improving consumer acceptability, while maintaining or enhancing nutritional quality” [2]. These cultures are carefully tailored to the specific requirements of the product and are selected for their ability to thrive under conditions such as controlled temperature, low pH, and high salt concentrations. Their enzymatic and antagonistic activities are also crucial for optimal performance [11]. Starter cultures facilitate microbial reactions such as acidification, catalase activity, and bacteriocin production and are typically added at concentrations of 5 to 8 CFU/g [2,12]. Starter cultures are generally classified into two main groups. The first group includes acidifying bacteria, primarily lactic acid bacteria (LAB). Common LAB species in starter cultures or isolated from traditional dry-fermented sausages include *Latilactobacillus sakei* (formerly *Lactobacillus sakei*), *Lactobacillus curvatus*, *Lactiplantibacillus plantarum* (formerly *Lactobacillus plantarum*), *Pediococcus pentosaceus*, and *Pediococcus acidilactici* [13,14,15,16]. LAB are essential for reducing pH, preventing microbial spoilage, and ensuring food safety. They also produce bacteriocins, which inhibit Gram-positive foodborne pathogens like *Listeria monocytogenes*, *Staphylococcus aureus*, and *Bacillus cereus* [17,18,19]. Additionally, LAB contributes to muscle protein coagulation, improving texture and flavor through enzyme activity such as cathepsin D-mediated proteolysis [2].

The second group includes coagulase-negative cocci (CNC), such as *Staphylococcus*, *Kocuria*, and *Micrococcus* species [11,17,18,19]. CNC stabilizes the red color of meat products by reducing nitrates to nitrites, facilitating nitrosomyoglobin formation, which imparts the characteristic cured meat color [11,20]. Their metabolic activities, including proteolysis and lipolysis, also enhance texture and sensory attributes [11,17,18,19].

The interplay of LAB and CNC significantly influences the texture, flavor, and overall sensory quality of fermented meat products. Factors such as recipe composition, processing conditions, and raw material properties further modulate these effects. LAB growth is particularly favored under anaerobic conditions, low pH, and high salt or sugar concentrations [21,22]. To optimize fermentation, mixed starter culture preparations are often employed, tailored to specific applications [11,23,24].

Honey, a product of nectar collected and processed by honeybees (*Apis mellifera*), contains carbohydrates (85–95%, primarily fructose and glucose), organic and amino acids, proteins, minerals, vitamins, and lipids. It also includes bioactive components such as tocopherol, ascorbic acid, flavonoids, and enzymes, which contribute to its antioxidant and antimicrobial properties [25]. Honey’s antimicrobial activity stems from its osmolarity, acidity, phenolic compounds, aromatic acids, and hydrogen peroxide [26]. Although honey promotes LAB growth, it inhibits the growth of other bacteria due to their sensitivity to its osmolarity and acidity [27].

The development of natural biopreservatives with antioxidants and antimicrobial properties to extend meat shelf life and prevent foodborne illnesses has garnered significant interest. While the antibacterial effects of honey in food systems have been previously studied [28], its influence on the physicochemical and technological properties, as well as consumer acceptance, of meat products remains largely unexplored.

This study aimed to analyze and compare the physicochemical, technological, and sensory characteristics of traditionally fermented sausages produced in Sokobanja, Southeastern Serbia, using pork meat. Bacterial starters isolated from other traditional meat products and varying concentrations of organic honey were incorporated. Additionally, this study assessed the presence and quantities of specific microbial groups during the 28-day ripening process to evaluate hygienic standards and food safety. Finally, it explored the potential of honey, used as a sugar source, in combination with starter cultures, to enhance the quality of dry-fermented sausages and their potential as functional foods.

## 2. Materials and Methods

### 2.1. Sausage Preparation

For sausage production, pork meat from the first and second categories (including cuts from the back, shoulders, and thighs) was used alongside a specified proportion of back fat, adhering to the traditional recipe of the manufacturer. The preparation of raw materials involved cutting the meat and fat into pieces and grinding them with a meat cutter featuring an 8 mm diameter. The ground meat was cooled to 0–5 °C for 24 h, after which a spice mixture was added, consisting of sugar (up to 0.4%), hot and sweet red pepper, garlic (up to 1%), and black and white pepper (up to 0.3%). After filling and squeezing, the sausages were smoked over beechwood smoke, at 16–24 °C, for at least 12–24 h, then subjected to drying and ripening under natural airflow conditions (usually in the attic) at an optimal temperature of −2 to 3 °C, without moisture or severe frost, for approximately 25 days, as long as low temperatures permitted preservation. Spontaneous fermentation played a key role in developing the characteristic microbiota and sensory properties of this traditional Sokobanja sausage from southeastern Serbia.

For the present study, a previously characterized bacterial culture was incorporated into the mixture, consisting of isolates from other traditional meat products. The starter culture included a combination of LAB and CNS: *Lactobacillus curvatus* sk1-8, isolated from dry-fermented Sokobanja sausage [29], known for its strong probiotic potential [30]; *Latilactobacillus sakei* IIb1, isolated from dry-cured sheep ham with good probiotic properties [31]; and *Staphylococcus xylosus* sk-Ios4, also from dry-cured sheep ham, exhibiting favorable technological properties [32]. Both Sokobanja sausage and dry-cured sheep ham are traditional Serbian meat products historically made without starter cultures [29,30,31,32]. These isolates were selected based on their origin, technological features, and probiotic attributes. Briefly, *L. curvatus* sk1-8 demonstrated good probiotic potential, including a high survival rate at low pH under simulated stomach and small intestine conditions, growth in the presence of high salt concentrations (up to 8%), growth in media containing 0.3% phenol, no detected synthesis of biogenic amines, high sensitivity to tested antibiotics, and strong antimicrobial activity [29,30]. *L. sakei* IIb1 exhibited tolerance to low pH and high salt, aggregation with enteropathogens, strong hydrophobicity, strong antimicrobial effects, no hemolysis, and sensitivity to antibiotics [31,32]. *S. xylosus* sk-Ios4 showed no hemolysis, growth across various pH values and salt concentrations, as well as proteolytic and lipolytic activity, indicating favorable technological properties [32]. The starter culture mixture, containing 1 × 10^9^ CFU/mL, was added at a ratio of 5 g per 50 kg of chopped meat.

In addition, organic sunflower honey (*Helianthus annuus Linn*.) from the Banat region in northeastern Serbia, renowned for sunflower cultivation, was also incorporated into the sausage mixture. This honey, thoroughly characterized by Milosavljević et al. [33], was confirmed to be free of *Lactobacillus* spp., which was crucial given the prior addition of starter cultures. Honey served as an additional sugar source for both autochthonous and starter cultures, acting as a prebiotic. Two sausage variants were tested based on the honey content: one with 0.2% (*w*/*w*) and another with 0.4% (*w*/*w*).

After mixing, the prepared meat with honey was allowed to stand for 4–5 h at 12–14 °C to enhance component integration. The mixture was then stuffed into pig intestines, approximately 32 mm in diameter. Following filling and squeezing, the sausages were smoked and dried under natural airflow conditions for 28 days.

This research involved three sausage variants. The first variant, serving as the control, was the original Sokobanja sausage, produced without honey (0%) and without mixed starter cultures. The second variant (test) included 0.2% honey and a mix of starter cultures, while the third variant (test) contained 0.4% honey and a mix of starter cultures.

The results of the test variants were compared to the control sausage. Ten sausages from each variant were analyzed, with all tests conducted in duplicate. Sampling for microbiological analysis, water activity (a_w_), and pH determination occurred on production days 0 (sausage stuffing), 3, 7, 14, and 28 during storage. Physicochemical, technological, and sensory analyses were performed at the end of the production process, on the 28th day of ripening.

### 2.2. Determination of Physicochemical Properties of Sausages

#### 2.2.1. Measurement of Water Activity (a_w_)

Water activity (a_w_) in sausages was measured using a Testo 650 device (Testo, Inc., Sparta, NJ, USA) equipped with a specialized probe for a_w_ measurements. The procedure involved filling a measuring cup to two-thirds of its capacity with coarsely chopped sausage and placing it in the probe’s measuring chamber. Measurements were conducted at a controlled room temperature (approximately 20 °C) until equilibrium was achieved, typically within two hours.

#### 2.2.2. Moisture Content Analysis

Moisture content was determined in accordance with the SRPS ISO 1442 [34] standard. A homogenized sample was thoroughly mixed with quartz sand and dried at 103 ± 2 °C until a constant weight was obtained. This process was performed using a TGA701 thermogravimetric analyzer (LECO Corporation, St. Joseph, MI, USA), with results expressed as a percentage (%).

#### 2.2.3. Determination of Total Ash Content

Total ash content analysis followed the SRPS ISO 936 [35] standard, which involved drying, carbonizing, and ashing the test sample at 550 ± 25 °C until a constant weight was reached. Moisture and ash content were simultaneously analyzed using the TGA701 thermogravimetric analyzer. The entire procedure required approximately five hours, with ash content reported as a percentage (%).

#### 2.2.4. Nitrogen and Protein Content Analysis

Non-Protein Nitrogen (NPN): NPN content was determined by analyzing the nitrogen in the filtrate obtained after protein precipitation with a 10% trichloroacetic acid (TCA) solution, using the Kjeldahl method [36]. A 10 g sausage sample was homogenized with 20 mL of 10% TCA for 60 s at 13,500 rpm using a T18 Basic Ultra-Turrax homogenizer (IKA-Werke GmbH & Co. KG, Breisgau, Germany). The mixture was allowed to precipitate for 2 h at 4 °C, followed by filtration. Nitrogen content in 10 mL of the filtrate was then measured using the Kjeldahl method, with results expressed as milligrams per kilogram of dry matter (mg/kg DM).

Total Protein Content: Protein content was calculated based on the total nitrogen (TN) determined by the Kjeldahl method [36]. The nitrogen content was multiplied by a conversion factor of 6.25. The process included sample digestion with concentrated sulfuric acid using copper (II) sulfate as a catalyst, followed by alkalization, ammonia distillation into boric acid, and titration with hydrochloric acid. Protein content was expressed as a percentage (%).

#### 2.2.5. Determination of Free Fat Content

Free fat content was measured using petroleum ether extraction with a Soxhlet apparatus, in accordance with SRPS ISO 1444 [37]. Results were expressed as percentage (%).

#### 2.2.6. Chloride Content (NaCl)

Total sodium chloride (NaCl) content was determined using the SRPS ISO 1841-1 standard [38]. Results were expressed as a percentage (%).

#### 2.2.7. TBARS (2-Thiobarbituric Acid-Reactive Substances) Test

The TBARS test, used to evaluate lipid oxidation, followed the methodology of Botsoglou et al. [39] with modifications by Šojić et al. [40]. Results were expressed as milligrams of malondialdehyde per kilogram of sample (mg MDA/kg).

### 2.3. Determination of Technological Properties of Sausages

#### 2.3.1. pH Determination

The pH of the samples was measured using a Testo 205 portable pH meter (Testo, AG, USA), which features a combined penetration tip and an integrated temperature probe.

#### 2.3.2. Color Determination

Color measurements were performed immediately after slicing the samples, following the procedure described by Tomović et al. [41]. The color parameters were expressed in the CIE Lab* system. The following parameters were calculated using standard mathematical formulas:

Hue − h = arctan (b*/a*)(1)

Color Saturation (C*) = ((a*)2 + (b*)^2^)^0.5^ (2)

Ten replicate measurements of both the surface and cross-sectional areas were conducted for each sample. The results were expressed as mean values.

### 2.4. Determination of Texturometric Properties of Sausage Varieties

Textural properties were evaluated using 1 cm thick slices of each sausage, with eleven slices analyzed per variety. Each slice was centrally positioned on the texturometer’s base table to ensure consistency during measurement. Firmness was assessed as the maximum peak force (N), while toughness was determined as the peak area, representing the work of shear (N/s). Hardness measured the force required for the first compression, and adhesiveness quantified the negative force needed to separate the compressing plunger from the sample. Springiness reflected the height recovery after the first compression, whereas cohesiveness represented the ratio of areas under the two compression curves. Chewiness was calculated as the product of hardness, springiness, and cohesiveness, and resilience (elasticity) described the sample’s ability to return to its original shape after deformation. All measurements followed the methodology described by Bourne [42].

### 2.5. Determination of Sensory Properties of Sausages

The sensory analysis was conducted at the accredited Laboratory for Food Product Testing, Faculty of Technology, University of Novi Sad (Accreditation Body of Serbia, accreditation number 01-059). Sensory analysis of traditional dry-fermented sausage with varying percentages of honey was performed by a panel of at least six trained assessors over multiple years. The evaluation employed an analytical descriptive scoring system ranging from 0 to 5, carried out in sensory analysis facilities designed in compliance with the SRPS EN ISO 8589 [43] standard. Each score corresponded to a specific quality level: a score of 0 indicated products with evident mechanical and/or microbiological defects, while a score of 1 denoted altered, atypical properties and an unacceptable product. A score of 2 reflected significant quality defects, 3 indicated noticeable defects, 4 represented slight deviations or minor quality imperfections, and 5 signified exceptional, typical sensory properties of optimal quality.

The sensory evaluation assessed various attributes, including external appearance and coating condition, cross-sectional appearance and composition, color consistency, odor, taste, texture, and juiciness. Each attribute was rated on the 0-to-5 scale, as described above, with the final score presented as the mean value of 20 repetitions.

### 2.6. Microbiological Analysis

Microbiological analysis was conducted on day 0 (sausage stuffing) and day 28 of the ripening process using standardized methods. The total count of aerobic mesophilic bacteria was determined according to SRPS EN ISO 4833-2 [44], while enterobacteria were quantified using ISO 21528-2 [45]. Coagulase-positive staphylococci were detected following SRPS EN ISO 6888-2 [46], and lactic acid bacteria (LAB) were identified using ISO 13721 [47]. Yeasts and molds were detected according to SRPS ISO 21527-1 [48], and *Listeria monocytogenes* was identified using ISO 11290-1 [49]. Species identification of *Salmonella* and *Shigella* was performed on SS agar (Torlak, Belgrade, Serbia), as described by Mokhtari et al. [50].

### 2.7. Statistical Analysis

All data were presented as means ± standard deviations, and statistical analysis was performed using Microsoft Excel (Redmond, Washington, DC, USA). A paired sample *t*-test (IBM SPSS Statistics 20) was used to compare control and test sausages.

## 3. Results

### 3.1. Physicochemical and Technological Properties of Sausages

The effect of the starter culture mix and different honey concentrations on the physicochemical characteristics of sausage variations at the end of ripening is shown in Table 1. The addition of 0.4% honey to sausages resulted in a significant (*p* < 0.05) decrease in total moisture content, total protein, nitrogen, and crude fat content. However, no significant differences were observed between samples in terms of total ash and chloride content (*p* > 0.05). As shown in Table 1, at the end of ripening, both sausage variants exhibited high fat content, with 30% in the sausage containing 0.2% honey and nearly 36% in the sausage with 0.4% honey, contributing to the sausages’ smooth texture.

Lipid oxidation was assessed by measuring TBARS levels (mg malondialdehyde/kg), as shown in Table 1. In this study, the addition of 0.4% honey significantly (*p* < 0.05) increased TBARS values compared to the 0.2% honey and control sausages.

**Figure 1:**
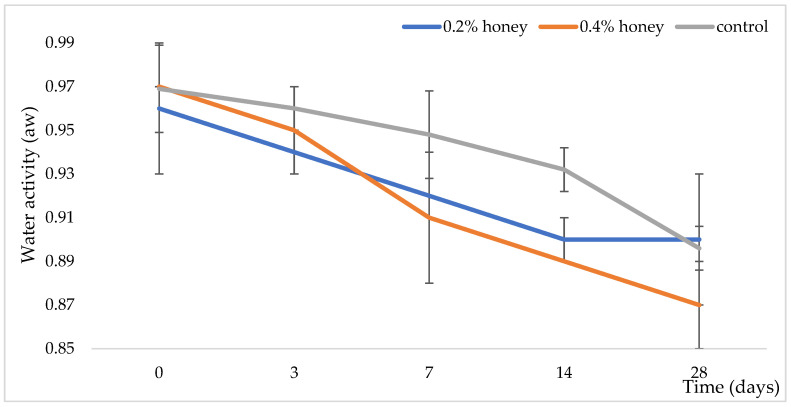
Changes in water activity (a*_w_*) throughout the ripening of examined variations of sausage.

As shown in Figure 1, the water activity (a_w_) values of the sausage varieties decreased over time. However, a noticeable difference was observed between the sausages with 0.2% and 0.4% honey. Specifically, the a_w_ value was lower in the sausage with 0.4% honey, while the sausage with 0.2% honey exhibited better aw values compared to the control sausage at the end of ripening. This decrease in a_w_ is primarily attributed to the moisture content (see Table 1).

**Figure 2:**
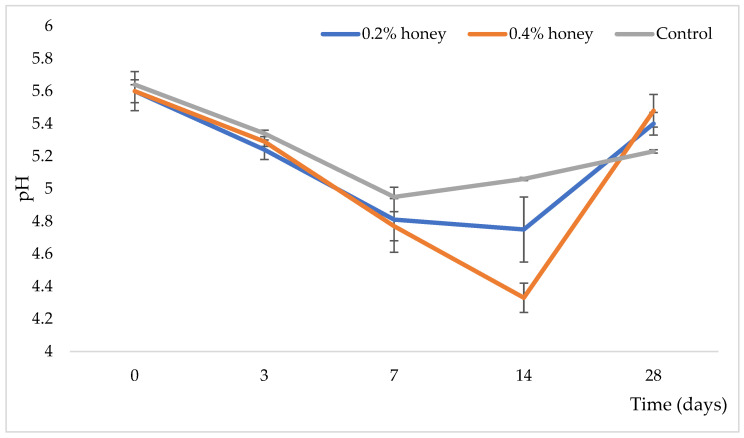
Changes in the pH values throughout the ripening of examined variations of sausage.

The different percentages of honey in the sausages did not significantly affect the pH values (Figure 2). During ripening, the pH decreased moderately, reaching its minimum on the 14th day of the process. Notably, on the 14th day, the pH value was lower in the sausage with 0.4% honey. All tested samples exhibited a decrease in pH until the 14th day, with sausages containing honey being more acidic compared to the control sausage. After this point, the pH values began to rise in all sausage varieties. However, by the end of the ripening period, the pH remained lower in the control sausage compared to those with honey.

The color parameters, including lightness (CIE L* value), redness (CIE a* value), yellowness (CIE b* value), color saturation (CIE C* value), and hue (h0 value), are presented in Table 2. Surface color, an important factor in sausage appearance, significantly impacts consumer acceptability. The sausages showed similar redness (a*) and color saturation (C*), while the cross-section displayed comparable lightness (L*) and redness (a*). The sausage with 0.2% honey appeared slightly lighter on the surface compared to the variant with 0.4% honey. Although the addition of honey did not notably affect redness, it did influence yellowness (b*). This change may be attributed to the inclusion of powdered red paprika, as evidenced by the relatively high a* and b* values.

The instrumental texture profile of the sausages, including firmness, toughness, hardness, adhesiveness, springiness, cohesiveness, chewiness, and resilience, was measured at the end of the ripening period, and the results are presented in Table 3. As anticipated, the texture analysis revealed differences among the sausage varieties. The addition of 0.2% honey to the sausage did not significantly affect firmness, adhesiveness, springiness, cohesiveness, or resilience compared to the control sausage. In fact, the values for springiness, cohesiveness, and resilience were lower in the sausage with 0.2% honey. Likewise, hardness, cohesiveness, and chewiness values were lower in the sausage with 0.2% honey compared to the control. On the other hand, the sausage with 0.4% honey displayed higher values for adhesiveness, cohesiveness, chewiness, and resilience compared to the other two sausage varieties.

### 3.2. Sensory Properties of Sausages

**Figure 3:**
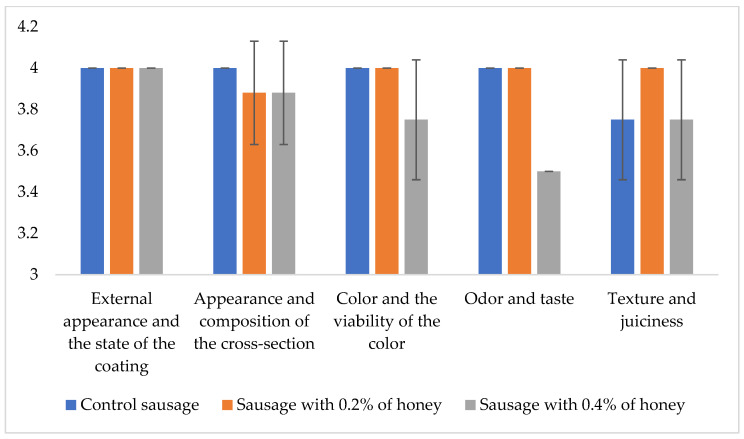
Sensory quality properties of sausage varieties (evaluated on a 0−5 scale).

The sensory panel results, presented in Figure 3, show that the addition of 0.4% honey significantly influences the sensory characteristics of the sausages, particularly in terms of color vibrancy, odor, taste, texture, and juiciness. Sausages with 0.2% honey displayed favorable sensory properties, closely resembling Sokobanja sausage. The trained sensory panel noted that these sausages had better texture and juiciness compared to the other two variations tested.

Sokobanja sausage was characterized by a brownish-red color, with visible white or light-yellow fat in the cross-section, a dry yet juicy texture, a smoky aroma, and a moderately to distinctly salted and dried profile, with a mildly tangy flavor. The sausages with 0.2% honey were wrinkled, varied in color and diameter, and exhibited a noticeable smoky aroma, although the honey flavor was not distinct. In contrast, the sausages with 0.4% honey were lighter and fattier, over-smoked, slightly bitter, and resembled barbecue sausages. Additionally, they showed mold on the surface.

### 3.3. Microbiological Analysis

**Figure 4:**
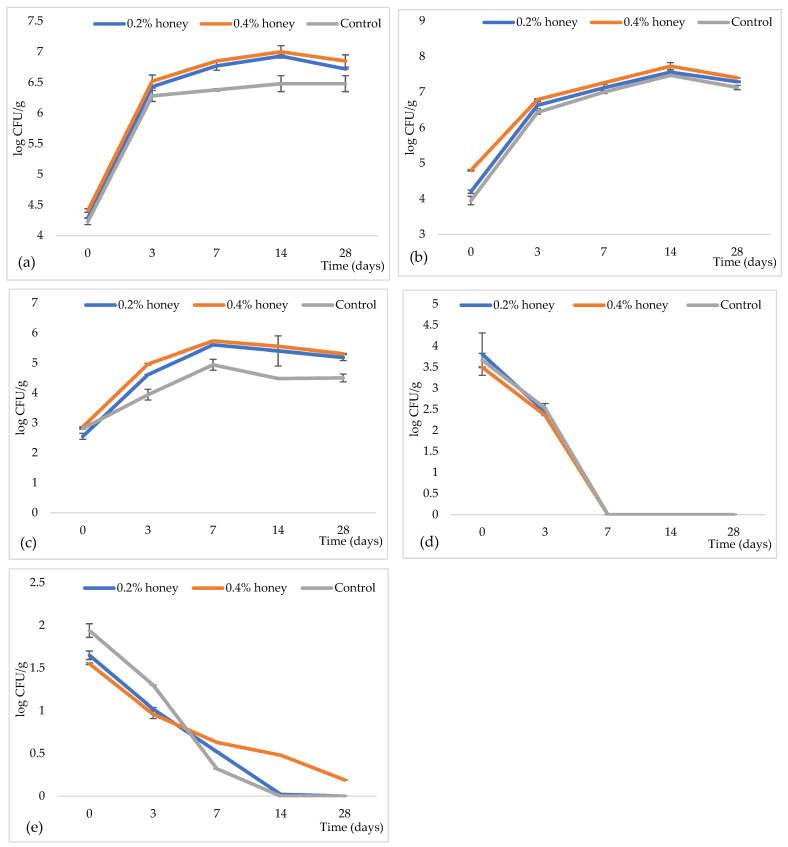
The dynamics of (**a**) total aerobic mesophilic bacteria, (**b**) total lactic acid bacteria, (**c**) total coagulase-negative staphylococci, (**d**) total enterobacteria, and (**e**) total yeasts and molds during the ripening of sausage varieties.

The results of the microbiological investigation of the three sausage varieties are presented in Figure 4. The initial count of aerobic mesophilic bacteria ranged from approximately 4.23 to 4.41 log CFU/g for all variants (Figure 4a), indicating that the raw mixture was produced under good sanitary conditions. The number of these bacteria increased until the 14th day of ripening in both sausage varieties with honey (6.93 and 7 log CFU/g). After the 14th day, their number began to decrease. In the control sausage, the number of aerobic mesophilic bacteria increased until the 3rd day of ripening, after which the number stagnated until the end of ripening.

The total viable LAB (Figure 4b) rapidly increased until the 14th day of ripening in all three tested varieties, reaching 7.47 log CFU/g in the control sausage, 7.56 log CFU/g in the sausage with 0.2% honey, and 7.73 log CFU/g in the sausage with 0.4% honey. By the 28th day of ripening, the number of LAB slightly decreased, but it remained higher in the sausage with 0.4% honey.

The total number of CNS (Figure 4c) increased until the seventh day of ripening. After the seventh day, the number of CNS decreased, likely due to the lowering of pH (Figure 2). The number of CNS was higher in the sausage varieties with honey compared to the control sausage. This increase is probably attributed to the presence of *S. xylosus* sk-Ios4 in the starter culture mixture used in the honey-containing sausages (see Section 2).

The number of enterobacteria (Figure 4d) was highest in the sausage stuffing on day 0, with counts of 3.67 log CFU/g in the control sausage, 3.50 log CFU/g in the sausage with 0.2% honey, and 3.81 log CFU/g in the sausage with 0.4% honey. After day 0, the number of enterobacteria rapidly decreased until the seventh day of ripening. A similar trend was observed for yeasts and molds, except for the sausage with 0.4% honey (Figure 4e). No bacteria from the genera *Salmonella* or *Shigella*, nor *L. monocytogenes*, were detected in any of the tested samples.

## 4. Discussion

Functional starter cultures for dry-fermented sausage production must meet several criteria related to health benefits, food safety, shelf life, technological efficiency, and cost-effectiveness. While probiotic bacteria are commonly used in dairy products, the food industry is increasingly focusing on the development of functional meat products with enhanced quality attributes [18]. Therefore, this study aimed to assess the impact of starter cultures and the addition of honey as a sugar source on the quality parameters of dry-fermented sausages, with potential applications as a functional food.

Organic sunflower honey, derived from the nectar of *Helianthus annuus* blossoms, is a natural, sustainably produced sweetener with a light to medium amber color and a distinctive sweet, floral, and slightly tangy flavor [33]. Produced under strict organic standards, it is free from synthetic pesticides, herbicides, and fertilizers, ensuring purity and environmental sustainability. Rich in natural sugars, antioxidants, vitamins, and minerals [51], it offers numerous health benefits, including antimicrobial and anti-inflammatory properties, making it a valuable addition to functional foods [25]. Its high glucose content contributes to smooth texture and rapid crystallization [33,51].

As a sugar source in food matrices, organic sunflower honey can support beneficial microbial growth, enhancing fermentation, flavor, and preservation while aligning with eco-conscious consumer preferences. LABs are known to thrive in high-sugar environments; however, excessive sugar content may negatively affect consumer acceptance [18,52]. For this reason, the authors investigated the impact of lower honey concentrations on the fermentation and ripening process of Sokobanja sausage. To the best of the authors’ knowledge, this is the first study exploring the role of organic sunflower honey in the fermentation of dry-fermented sausages.

Additionally, the authors added isolated bacterial cultures (*L. curvatus* sk1-8, *L. sakei* IIb1, and *S. xylosus* sk-Ios4) to the chopped meat, selected based on their origin, technological attributes, and probiotic characteristics [29,30,31,32], to create a functional food product with high consumer acceptance. The addition of 0.4% honey and the starter mix culture resulted in a lower percentage of moisture, total nitrogen, and total protein content, while leading to a higher percentage of free fat and TBAR values (see Table 1). At the end of the ripening period, the sausage with 0.2% honey exhibited better a_w_ values compared to the control sausage. This is likely due to the moisture content of the honey [33], the optimal concentration of honey added, and optimal fermentation conditions related to the autochthonous microbiota and the added starter cultures.

The pH of fermented sausage is a critical factor influencing its fermentation process, texture, flavor development, and microbial stability [11]. According to Grujović et al. [18], the pH decrease in dry-fermented sausages results from spontaneous fermentation driven by autochthonous (non-starter) lactic acid bacteria (NSLAB). This pH drop is crucial for controlling microbial growth, enhancing flavor, and ensuring sausage safety [17,18,19]. In our investigated sausage varieties, which contain both NSLAB and added starter cultures (see Section 2), the combination of starter and non-starter LAB accelerates the acidification process compared to control sausage. The subsequent pH increase during the later stages of ripening is likely due to a reduction in lactic acid content rather than the formation of low molecular weight nitrogen compounds [53].

The rate and extent of pH change are influenced by factors such as the type of starter cultures, initial meat pH, sugar content, and environmental conditions during fermentation (e.g., temperature and humidity) [54]. A well-controlled pH reduction is essential for achieving a balanced flavor profile, desirable texture, and microbial safety in the final product [17,18,19].

Typically, the pH of dry-fermented sausages starts in the range of 5.5–6.0 and decreases to a final pH of 4.8–5.2 by the end of fermentation [55], consistent with the results of our investigation. This acidification stabilizes the product by inhibiting spoilage and pathogenic microorganisms, such as *Salmonella* spp. and *Listeria monocytogenes* [17,18,19]. Furthermore, the pH reduction denatures muscle proteins, enhancing the texture and cohesiveness of the sausage [2].

As suggested by Cachaldora et al. [56], color is one of the most important parameters influencing consumer perception and acceptability. The color of sausage is influenced by several factors, including the natural color of the meat, the presence of additives (such as spices, salts, and curing agents), and changes that occur during fermentation and drying [57]. The color of dry-fermented sausages can change throughout the ripening and fermentation process. During fermentation, LAB and other microorganisms produce lactic acid, which lowers the pH and causes protein denaturation, potentially altering the sausage’s color [58,59]. Our investigation indicated that the addition of honey did not significantly affect redness but did influence yellowness (b*). This effect may be attributed to the inclusion of powdered red paprika, as indicated by the relatively high a* and b* values. Ikonić et al. [5] associated the pronounced red (a*) and yellow (b*) coloration on the cut surfaces of Perovská klobása with the use of a substantial amount of red-hot paprika powder. Similar findings were reported by Stajić et al. [8] in their study on Sremska sausage.

The pH value can influence hardness and chewiness (lower pH typically results in higher hardness and chewiness). However, in this case, all sausage varieties exhibited similar pH values at the end of production, except for samples with 0.4% honey. Our results indicated that the sausage with 0.4% honey had higher hardness and chewiness values compared to the sausage with 0.2% honey at the end of the ripening process. Thus, the addition of a higher concentration of honey likely affects the hardness and chewiness of the sausage, as the pH values were consistent across the varieties at the end of production (Figure 2). Moisture and fat content can also impact the texture of fermented sausages—higher moisture and fat levels generally result in lower hardness and chewiness [60]. However, the sausage with 0.2% honey showed better hardness and chewiness properties compared to the control sausage, suggesting that this concentration of honey, along with other factors, positively influenced the texture properties of the sausage.

Adding sugar to sausage formulations lowers the pH below the isoelectric point of myofibrillar proteins, resulting in higher hardness and chewiness values [61]. This may be due to reduced moisture content during drying and an increase in fat concentration. Our investigation indicates that adding 0.4% honey negatively impacts the sausage’s texture profile. Conversely, 0.2% honey positively affects hardness, cohesiveness, and chewiness, with these values being lower in the sausage with 0.2% honey compared to the control sausage. This effect may be due to the texture of honey [33] and the higher free fat content in sausages with 0.2% honey (see Table 1).

Higher adhesiveness values were observed in samples with 0.4% honey. This could be due to factors such as formulation, moisture content, fat, protein interactions, and microbial fermentation, all of which could increase probe adhesion to the analyzer’s working part. During fermentation, lactic acid production lowers the pH, affecting protein breakdown and sausage texture, resulting in a firmer or stickier product. Moisture and salt may enhance protein or hydrocolloid solubility, forming a sticky film on the sausage surface. This film increases the work required to overcome adhesion forces during contact with the analyzer’s working part [62].

Cohesiveness indicates how the sample withstands a second compression (biting) relative to its behavior during the first compression. Cohesiveness values range from 0 to 1 [63]. Sausages with 0.4% honey exhibited higher cohesiveness values compared to the other two varieties.

Chewiness, a key texture property of dry-fermented sausages, is often considered an important indicator of product quality and consumer acceptability. It is calculated as the product of hardness, cohesiveness, and springiness, representing the work required to chew the sample [63]. This parameter reflects the product’s structural and mechanical properties and its behavior during consumption. Based on the results presented in Figure 2, we conclude that the sausage with 0.2% honey exhibited the best chewiness characteristics.

As mentioned, the values for springiness, cohesiveness, and resilience were lower in the sausage with 0.2% honey compared to the control sausage, but the differences were not statistically significant (see Table 3). The inclusion of a lower concentration of honey may have contributed to these minor textural changes due to its hygroscopic nature [33,51], which affects moisture balance, and its influence on protein denaturation and fermentation processes. While honey enhances flavor and microbiological safety, it may slightly decrease the elasticity and cohesion of the sausage compared to the control. Additionally, sensory panel results (Figure 3) indicated that sausages with 0.2% honey exhibited favorable sensory properties, closely resembling those of the Sokobanja sausage, which served as the control.

The reduction in enterobacteria to undetectable levels after the 7th day of ripening implies significant improvements in both the safety and shelf life of the product. This reduction is consistent with previous findings, which demonstrate the dominance of LAB and a sharp decline in enterobacteria populations during sausage fermentation [64]. Enterobacteria are often associated with foodborne illnesses, spoilage, and contamination, so their elimination reduces the risk of pathogen-related safety concerns [64,65].

The increased LAB count, driven by the addition of honey and probiotics and its enhancement of LAB growth, plays a critical role in the fermentation process. LAB produces metabolic byproducts, such as organic acids and bacteriocins, which lower the pH and inhibit the growth of spoilage microorganisms and pathogens [18]. This creates an environment less conducive to microbial spoilage, thereby extending the shelf life of the product while ensuring its safety for consumption [64].

Overall, the results of this study indicate that organic sunflower honey has a favorable impact on the sensory, nutritional, and textural properties of dry-fermented sausage. A 0.2% honey addition resulted in a more desirable product with optimal flavor, texture, and functional characteristics. Furthermore, the use of starter cultures (*L. curvatus* sk1-8, *L. sakei* IIb1, and *S. xylosus* sk-Ios4) contributed to the fermentation process and the creation of a product with health benefits, in line with the growing demand for functional foods.

## 5. Conclusions

The results of this investigation indicate that the addition of starter cultures and sunflower honey significantly influenced the physicochemical and microbiological properties of the sausage. Specifically, incorporating starter cultures and 0.2% honey into the sausage mixture positively impacted the hardness, cohesiveness, and chewiness values, outperforming the control sausage. These sausage samples also received high scores in the consumer acceptance test. The sugars in the honey supported the growth of autochthonous and starter LAB and CNS cultures during the first 14 days of ripening, while the growth of enterobacteria, yeasts, and molds rapidly declined after the 7th and 14th days. This was likely due to the reduction in pH caused by lactic acid production by LAB.

As noted, the typical ripening period for Sokobanja sausage is around 25 to 28 days. However, the ripening process of the examined sausages was accelerated by adding starter cultures and honey, as the starter cultures enhanced LAB activity. The combination of honey and starter cultures contributed to a faster pH drop, creating a favorable environment for the growth of beneficial microorganisms, which, in turn, helped improve the texture and safety of the sausage. This result aligns with previous studies showing that honey, particularly its sugar content, can promote microbial fermentation in meat products, leading to improved product characteristics.

Overall, this study positions these sausages as a functional food enriched with probiotics LAB and natural ingredients, offering potential health benefits such as improved gut health and antimicrobial properties. The antimicrobial properties of the mixed starter cultures, combined with honey, further emphasize the low risk of pathogen growth and ensure product safety. Additionally, the antioxidant properties of honey position the product as a source of natural antioxidants, helping to combat oxidative stress and contribute to overall health. Honey, known for its prebiotic properties, also supports healthy gut flora, making the product beneficial for digestion. Furthermore, since honey and starter cultures are natural ingredients, the sausages can be marketed as free from artificial preservatives, offering a clean-label option for health-conscious consumers. The improved sensory qualities of the sausages, including enhanced flavor and texture, provide a competitive edge, as the product combines health benefits with great taste.

Nonetheless, additional research is necessary, particularly on the selection of appropriate carriers for the targeted delivery of probiotic bacteria to specific sites within the gastrointestinal tract, ensuring high cell viability in the food matrix up to the point of consumption. This would enhance the potential of these sausages as a functional food product that can provide health benefits beyond basic nutrition. Additionally, the potential health benefits of this sausage, especially regarding gut health, immune modulation, and antimicrobial properties, require further investigation. Overall, this study opens up new possibilities for developing functional meat products enriched with beneficial bacteria and natural ingredients like honey, which could offer health-promoting effects while maintaining high sensory appeal.

## Figures and Tables

**Table 1:** Effect of starter mix culture and addition of honey on physicochemical properties of sausages.

Chemical Characteristics	Type of Sausage		
	Control	0.2% of Honey	0.4% of Honey
Total moisture content (%)	38.3 ± 0.60 ^a^	38.5 ± 0.10 ^a^	34.0 ± 0.09 ^b^
Total ash content (%)	4.5 ± 0.10 ^a^	4.5 ± 0.14 ^a^	4.5 ± 0.03 ^a^
Total nitrogen content (mg/kg)	28.62 ± 0.34 ^a^	30.50 ± 0.10 ^a^	18.75 ± 0.25 ^b^
Total protein content (%)	23.4 ± 1.40 ^a^	22.4 ± 0.23 ^a^	19.4 ± 0.13 ^b^
Free fat content (%)	34.7 ± 0.80 ^a^	30.10 ± 0.05 ^b^	35.90 ± 0.11 ^a,c^
Total chloride content (%)	2.8 ± 0.10 ^a^	2.71 ± 0.01 ^a^	2.63 ± 0.03 ^a^
TBAR (mg MDA/kg)	0.299 ± 0.016 ^a^	0.350 ± 0.012 ^b^	0.397 ± 0.011 ^c^

Values are presented as mean ± SD; means ± SD with different superscript letters in the same row differ significantly (*p* < 0.05).

**Table 2:** Effect of different concentrations of honey on the color parameters of sausage.

Color Parameters	Surfaces of Sausages			Cross-Sections of Sausages		
	Control	0.2% of Honey	0.4% of Honey	Control	0.2% of Honey	0.4% of Honey
CIE L* value	35.94 ± 2.24 ^a^	35.79 ± 1.31 ^a^	37.49 ± 2.41 ^b^	49.67 ± 2.62 ^a^	50.26 ± 3.52 ^a^	51.01 ± 3.66 ^a^
CIE a* value	15.26 ± 0.42 ^a^	15.51 ± 0.51 ^a^	16.11 ± 0.93 ^a^	17.99 ± 1.78 ^a^	18.33 ± 1.83 ^a^	18.91 ± 1.49 ^a^
CIE b* value	9.22 ± 0.98 ^a^	14.81 ± 1.45 ^b^	16.18 ± 2.88 ^c^	10.64 ± 1.18 ^a^	15.43 ± 2.07 ^b^	18.62 ± 1.61 ^c^
CIE C* value	21.88 ± 1.76 ^a^	21.46 ± 1.23 ^a^	22.17 ± 3.30 ^a^	20.26 ± 1.36 ^a^	23.98 ± 2.61 ^b^	26.57 ± 1.79 ^c^
h^0^ value	44.32 ± 1.87 ^a^	43.55 ± 2.51 ^a^	46.73 ± 2.71 ^b^	40.31 ± 1.60 ^a^	39.99 ± 2.14 ^a^	44.54 ± 2.73 ^b^

Values are presented as mean ± SD; means ± SD of the surface/cross-section of sausage with different superscript letters in the same row differ significantly (*p* < 0.05). The surface and the cross-section are compared separately.

**Table 3:** Texture profile of the tested sausages.

Texture Properties	Type of Sausage		
	Control	0.2% of Honey	0.4% of Honey
Firmness (N)	13.98 ± 1.67 ^a^	15.74 ± 2.65 ^a^	9.39 ± 1.83 ^b^
Toughness (N/s)	93.14 ± 12.42 ^a^	114.27 ± 15.07 ^b^	67.68 ± 17.18 ^c^
Hardness (N)	18,981.66 ± 1479.23 ^a^	17,454.86 ± 3921.45 ^b^	19,191.99 ± 2059.07 ^a,c^
Adhesiveness (N/s)	−107.49 ± 69.14 ^a^	−110.47 ± 61.38 ^a^	−403.88 ± 743.23 ^b^
Springiness (ratio)	0.722 ± 0.036 ^a^	0.713 ± 0.058 ^a^	0.753 ± 0.015 ^a^
Cohesiveness (ratio)	0.586 ± 0.098 ^a^	0.523 ± 0.108 ^a^	0.604 ± 0.058 ^b^
Chewiness (N)	7128.58 ± 1287 ^a^	6469.36 ± 1801.55 ^b^	8749.92 ± 1412.25 ^c^
Resilience (ratio)	0.229 ± 0.089 ^a^	0.222 ± 0.047 ^a^	0.254 ± 0.022 ^b^

Legend: means ± SD with different superscript letters in the same row differ significantly (*p* < 0.05).

## Data Availability

The original contributions presented in the study are included in the article, further inquiries can be directed to the corresponding author/s.
